# Hemostatic Cryogels Based on Oxidized Pullulan/Dopamine with Potential Use as Wound Dressings

**DOI:** 10.3390/gels8110726

**Published:** 2022-11-09

**Authors:** Raluca Ioana Baron, Ioana A. Duceac, Simona Morariu, Andra-Cristina Bostănaru-Iliescu, Sergiu Coseri

**Affiliations:** 1 Department of Polyaddition and Photochemistry, “Petru Poni” Institute of Macromolecular Chemistry, 41A Grigore Ghica Voda Alley, 700487 Iasi, Romania; 2 Department of Natural Polymers, Bioactive and Biocompatible Materials, “Petru Poni” Institute of Macromolecular Chemistry, 41A Grigore Ghica Voda Alley, 700487 Iasi, Romania; 3 Laboratory of Antimicrobial Chemotherapy, “Ion Ionescu de la Brad” University of Life Sciences, 8 Mihail Sadoveanu Alley, 700489 Iasi, Romania

**Keywords:** Schiff base, acetal bonds, self-healing, mechanical stability

## Abstract

The impetus for research into hydrogels based on selectively oxidized polysaccharides has been stimulated by the diversity of potential biomedical applications. Towards the development of a hemostatic wound dressing in this study, we creatively combined the (hemi)acetal and Schiff base bonds to prepare a series of multifunctional cryogels based on dialdehyde pullulan and dopamine. The designed structures were verified by NMR and FTIR spectroscopy. Network parameters and dynamic sorption studies were correlated with environmental scanning microscopy results, thus confirming the successful integration of the two components and the opportunities for finely tuning the structure–properties balance. The viscoelastic parameters (storage and loss moduli, complex and apparent viscosities, zero shear viscosity, yield stress) and the structural recovery capacity after applying a large deformation were determined and discussed. The mechanical stability and hemostatic activity suggest that the optimal combination of selectively oxidized pullulan and dopamine can be a promising toolkit for wound management.

## 1. Introduction

Hydrogels are regarded as the most promising contender among the many different materials used for wound dressing because of their one-of-a-kind qualities, including their soft nature, great flexibility, a tremendous capacity for retaining water, and high level of biocompatibility [1,2]. Hydrogels that can self-heal after being damaged have recently attracted a lot of interest because of their ability to function similarly to human tissues in this regard [3,4,5,6]. Because of their rapid in situ formation, dynamic reversibility, and environmental pH responsiveness, the Schiff base bond has been regarded as a good choice among the various methods of fabrication available. For these reasons, it can be successfully chosen to fabricate self-healing hydrogels that can be most suitable for bleeding wound management [2,7,8,9]. However, due to their poor mechanical resistance and tissue adhesion performance, Schiff base cross-linked self-healing hydrogels have a limited impact on wound healing on their own. Consequently, it is still difficult to achieve high mechanical and adhesive strength without sacrificing self-healing capability and biocompatibility.

In recent years, it has become known that polysaccharide derivatives that include aldehyde groups may function very well as efficient cross-linking reagents for polymers that contain amino groups. Synthesis and application of dialdehyde alginate [10,11,12], dialdehyde cellulose [13,14,15], dialdehyde dextran [16], dialdehyde carboxymethyl cellulose [17,18,19], and dialdehyde xanthan gum [20] have been obtained in the past aiming to fabricate cross-linked films, fibers, and cryogels. These materials were primarily designed for use in food or biomedical applications.

Another candidate very well suited for preparing physically and/or chemically cross-linked hydrogels is pullulan. Multiple species of the bacterium *Aureobasidium* produce pullulan, a linear repeating polysaccharide that is both water-soluble and biodegradable [21]. It is made up of hundreds of glucose units that are repeated, and each one is connected to the next by -1,6-, and -1,4-glycosidic linkages. Pullulan possesses outstanding biological and physical features, including the qualities of being atoxic, edible, biocompatible, biodegradable, and water-soluble, in addition to possessing adhesive qualities [22,23]. Because of the presence of a variety of glycosidic linkages, it has physicochemical characteristics that are unique [24]. Pullulan is capable of undergoing a variety of chemical transformations, which may result in the formation of derivatives with significantly altered structures and characteristics. One of the reactive derivatives that may be formed from the periodate [25,26] or TEMPO [27] selective oxidation of pullulan is a compound known as pullulan dialdehyde, which contains polyaldehyde structures. Due to the presence of three distinct anhydroglucose rings in the repeating unit of pullulan, oxidation with periodate will produce many distinct forms of dialdehyde compounds [25]. These active groups have the potential to react with the free amino groups in a way that is analogous to that of glutaraldehyde, alginate dialdehyde, dialdehyde cellulose, dextran dialdehyde, oxidized xanthan gum, and other related compounds. In the past, pullulan was combined with sodium periodate (NaIO_4_) and human-like collagen at the same time to produce hydrogels for use in skin restoration [19]. In more recent times, it has been observed that oxidized pullulan that contains carboxyl groups may speed up the process of network formation in PVA hydrogels [22].

Given the experience of our research group in obtaining polysaccharides functionalized using selective oxidation methods, particularly pullulan, and their further use for preparing hydrogels [22,28,29,30], we propose in this study a new polymeric system. We hypothesized that periodate-oxidized pullulan can form stable hydrogels based on (hemi)acetal bonds and additional dopamine can be incorporated with success by interacting with the aldehyde groups, improving the stability of the networks. This polysaccharide-based material should exhibit self-healing behavior, good swelling degree and excellent hemostatic activity, and therefore specific characterizations were performed.

## 2. Results and Discussion

Polymeric materials processed in the form of cryogels can be obtained by various strategies. Physical and chemical cross-links have been explored in the literature with significant success in finely tuning network parameters and properties [28]. In this context, we hypothesized that stable networks can be obtained by combining dialdehyde pullulan and dopamine, aiming to create a material suitable for dressing in the management of bleeding wounds. Such hydrogels pose many benefits associated with them, including the fact that the raw materials are cost-effective, nontoxic and harmless, and the preparation process is straightforward.

In this study, dopamine was not immobilized on the polysaccharide through carbodiimide chemistry (leading to an amide bond) or by reduction of the imine bond to a secondary amine with NaBH_4_. Instead, the Schiff base bond was successfully used, since the reaction occurs readily.

**Figure 1:**
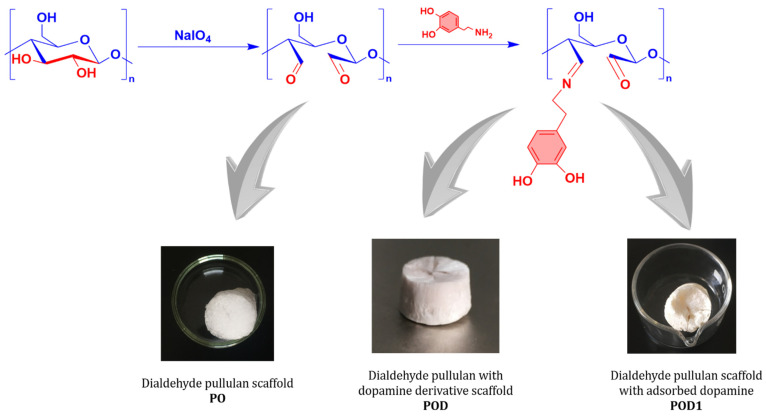
Schematic illustration of the conceptual reaction steps involved in the preparation of oxidized pullulan–dopamine cryogels and optical images of the resulting materials.

The designed reaction pathway is depicted in Figure 1. First, pullulan was selectively oxidized in the presence of sodium periodate. The reaction resulted in the formation of two new aldehyde groups at the C2 and C3 atoms of the anhydroglucose ring, simultaneously with the break of the C–C bond between these atoms. The oxidized derivative was freeze-dried as the control sample PO and further used to make dialdehyde pullulan–dopamine cryogels. Subsequently, two methods were employed to test the most reliable way for obtaining dialdehyde pullulan–dopamine cryogels. These methods were both based on the chemical interaction between the aldehyde moieties on the pullulan backbone and the amino groups in the dopamine structure. One path that was explored to prepare the POD sample (Figure 1) was to perform the reaction in a dialdehyde pullulan solution, where dopamine was added. The resulting pullulan derivative grafted with dopamine was subjected to freeze-drying. In contrast, the other path that was explored to prepare the POD1 sample was to perform the Schiff base reaction using an already freeze-dried dialdehyde pullulan scaffold that was immersed in a dopamine solution. Therefore, the compound was adsorbed into the porous network and interacted with the aldehyde groups available on the surface of the pores.

UV-vis measurements were performed on both samples to determine the content of dopamine attached to pullulan by the Schiff base reaction. The POD sample had 20% dopamine attachment compared to the POD1, in which only 1.14% of the dopamine was retained inside the polysaccharide network.

### 2.1. Structural Characterization of the Pullulan–Dopamine Cryogels

Several types of interactions are possible and probable inside the networks based on oxidized pullulan alone and oxidized pullulan–dopamine, respectively. These may occur in distilled water solutions and also during the freeze-drying process. In the former case, the dialdehyde polysaccharide obtained by oxidation with periodate rearranges spontaneously and can easily lead to the formation of intra- and inter-chain acetals and hemiacetals, thus resulting in cross-linking [31,32]. In the latter case, the addition of dopamine into the polysaccharide network enables the formation of the following interactions: (i) numerous hydrogen bonds between the hydroxyl groups existent in catechol and anhydroglucose rings; (ii) π–π interactions by stacking; (iii) acetal and hemiacetal bonds between the hydroxyl groups existent in catechol and the aldehyde moieties in pullulan. In both cases, the freeze-drying process is favorable to the formation of additional H-bonds. These assumptions were analyzed by means of NMR and FTIR spectroscopy in the following sections.

#### 2.1.1. NMR Spectra Analysis

**Figure 2:**
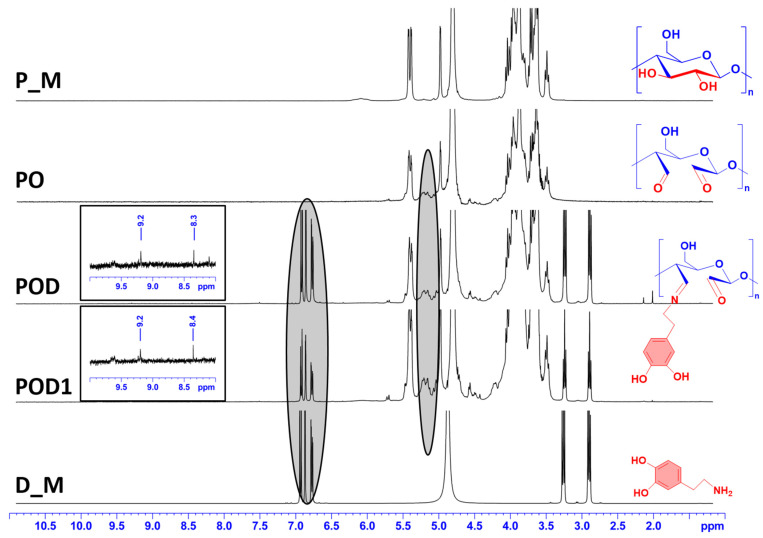
^1^H-NMR spectra recorded for pullulan, dopamine and the three types of cryogels.

The first method used to confirm the chemical modification of pullulan and to validate the accomplishment of successful network formation in the cryogels was 1H-NMR spectroscopy. Spectra were recorded for all samples, namely pullulan (P_M), periodate oxidized pullulan (PO), the dialdehyde pullulan–dopamine cryogels (POD and POD1), and dopamine (D_M), see Figure 2.

The pullulan spectrum (P_M) contains typical peaks for the polysaccharide structure. In contrast to that, peaks between 5 and 5.5 ppm can be seen in the spectra of all three cryogels (PO, POD, and POD1). These peaks can be correlated with the formation of hemiacetal clusters between the polysaccharide backbone of different chains [33]. In the spectrum recorded for the D_M sample, the aromatic protons can be associated with the peaks between 6.6 and 6.8 ppm. The signals detected at 2.9 and 3.3 ppm correspond to the methylene hydrogen that is located close to the benzene ring and the hydrogen that is located adjacent to the amino group in the structure of dopamine, respectively [34]. It is readily observed that all of the characteristic signals seen in the D_M spectrum are also found in the spectra of POD and POD1 cryogels. In addition, new peaks can be observed in the spectra of POD and POD1 samples at 8.3/8.4 and 9.2, attributed to the imine proton [35] and the aldehyde proton [36], respectively. This is the most conclusive evidence that dopamine was effectively grafted onto the oxidized pullulan chains, independently of the incorporation method used when preparing the material.

#### 2.1.2. FTIR Spectra Analysis

**Figure 3:**
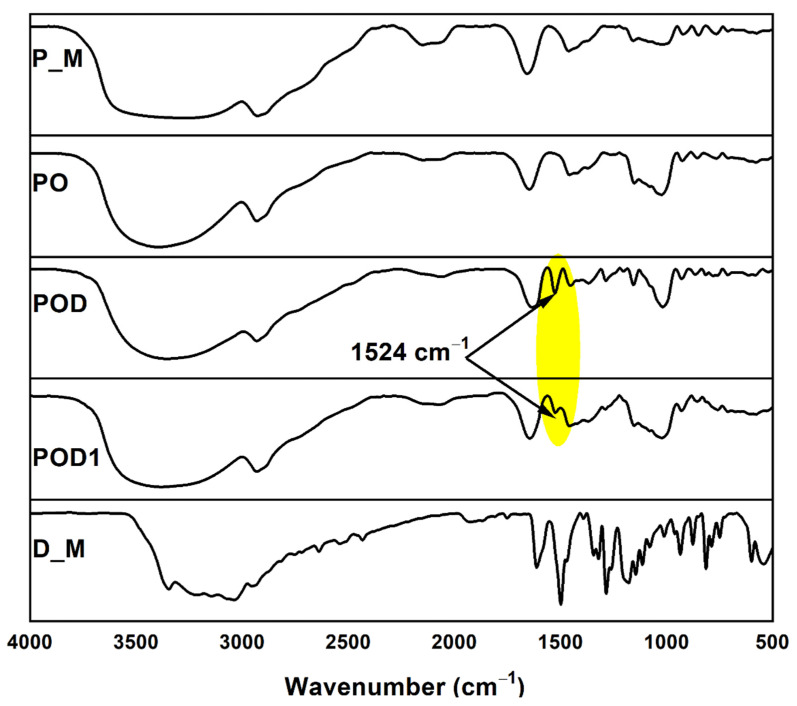
FTIR spectra of the raw polysaccharide, dopamine and the prepared cryogels.

For an in-depth characterization of the designed materials, FTIR spectroscopy was used. Figure 3 displays the FTIR spectra of the raw polysaccharide (P_M) and dopamine (D_M) samples, as well as the three types of cryogels (PO, POD, and POD1), all recorded in the range between 4000 and 500 cm^−1^.

The pullulan sample exhibited an FTIR spectrum that is characteristic for the class of polysaccharide and includes the following features: a broad band in the 3600–3000 cm^−1^ region due to the OH stretching vibration; a band at 2930 cm^−1^ assigned to the C–H stretching vibration; the bands around 1460 cm^−1^ can be assigned to the symmetric bending vibration of the CH_2_ groups; finally, the band at 924 cm^−1^ is corresponding to C–O–C stretching of α-(1→4)-glycosidic bonds [26].

In the D_M spectrum, the stretching vibrations of the C=C aromatic ring and the N–H bending are the strongest and they are overlaid in the absorption band recorded at 1614 cm^−1^ [37]. Moreover, the stretching vibrations of the O–H and C–H bonds can be correlated to the peaks at 3354 and 2942 cm^−1^ [38]. The band at 1179 cm^−1^ is representative of the C–O stretching vibrations.

Notably, two major conclusions must be drawn regarding the spectra recorded for the cryogel samples. On the one hand, the distinctive peaks of pullulan were preserved in their entirety, which demonstrates that pullulan was the primary component of the cryogel structure. On the other hand, in the spectra of POD and POD1 cryogels, there is a new peak at 1524 cm^−1^ compared to the PO spectrum, which is characteristic of/associated with the C–N bonds [39,40] and strongly suggests that dopamine was successfully introduced into the hydrogel network.

### 2.2. Internal Morphology and Network Parameters

#### 2.2.1. Environmental Scanning Microscopy (ESEM) Studies

**Figure 4:**
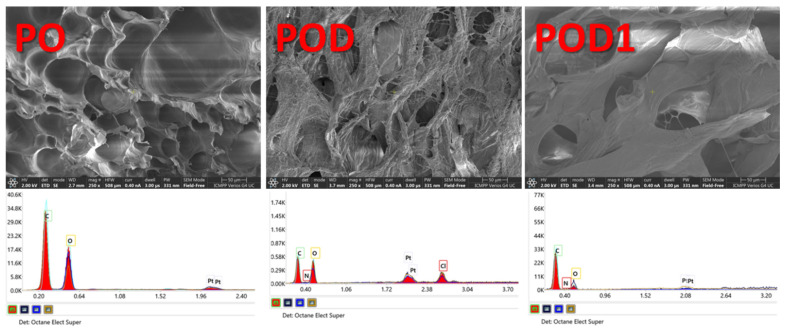
SEM micrographs of the internal morphology recorded for the three types of hydrogels and the corresponding EDAX spectra.

Internal morphology is a key feature in understanding the behavior of a material, especially in 3D networks, such as freeze-dried hydrogels. Therefore, SEM images and EDAX spectra were recorded for all three cryogels (Figure 4). The micrographs reveal a porous structure with interconnected pores of variable diameter for all samples. However, multiple differences can be noted. The pullulan control sample, PO, has pores with smooth surfaces. When dopamine is introduced into the oxidized pullulan solution, the interactions lead to the formation of a sponge with very rough internal surfaces and filiform cross-links inside the volume of the pores. By comparison, dopamine adsorption from the solution into the dialdehyde pullulan freeze-dried network led to a material with an intermediate internal appearance. The pore surfaces in the POD1 sample are uneven with filiform protuberances and, to a lesser degree than POD, with thread-like cross-links inside the pores.

The EDAX spectra confirm once more the addition of dopamine. As a result of introducing this compound, a supplementary peak can be observed in the spectra of POD and POD1, which is attributed to the presence of nitrogen on the surface subjected to the analysis. In addition, the intensity of the peaks specific for C and O dropped correspondingly. Therefore, dopamine was successfully included in the polysaccharide matrix with nitrogen levels of 3.1% and 2.4%, respectively for POD and POD1.

#### 2.2.2. Porosity and Density of the Cryogels

**Figure 5:**
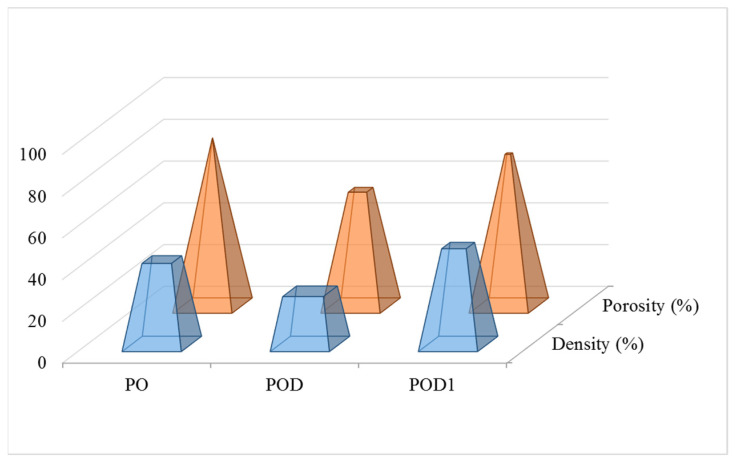
Density and porosity levels recorded for the pullulan–dopamine cryogels.

Hydrogels are polymeric networks with porosity and density depending on the type of polymer, type of cross-links, cross-link density, pore size and tortuosity etc. In this case, we hypothesized that periodate-oxidized pullulan can form hydrogels based on physical junctions and entanglements, associated with a sufficient number of hemiacetal interchain interactions. The presence of dopamine entrapped into the pullulan matrix can increase the number of interactions and change the network properties, e.g., the mesh size, distance between cross-links and cross-link density. Therefore, the cryogels were measured in terms of porosity and density. The results are illustrated in Figure 5. As expected, PO has the highest porosity, 80.41%, followed by POD1 (72.76%) and POD (55.43%). These results illustrate that inside the pores of the oxidized pullulan cryogel the aldehyde groups are oriented in such a way that they allow the adsorption of dopamine. In addition, the large molecules of dopamine fill the pore space and form novel, supplementary interactions. Interestingly, the network density is the greatest for the POD1 (47.1%), closely followed by the PO cryogel (40.4%). Meanwhile, the lowest value was recorded for the POD sample (25.2%), which correlates with the porosity data and the SEM images.

### 2.3. Dynamic Water Vapor Sorption Efficiency

**Figure 6:**
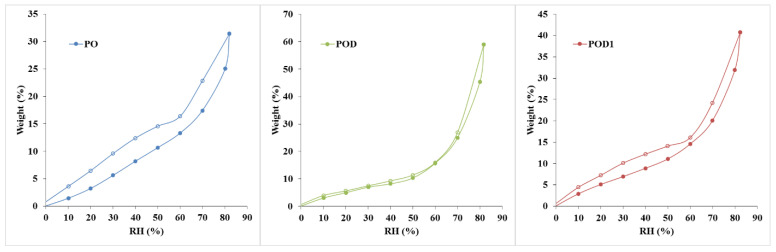
Water adsorption isotherm curves (with full circles) and hysteresis cycles (desorption curves with empty circles) plotted for the oxidized pullulan–dopamine hydrogels.

Dynamic vapor sorption measurements are widely used to determine the surface area and pore size distribution of various materials. Especially in hydrogels, it is critical to collect information about their interaction with water molecules. The complexity of such investigations is entailed by the fact that numerous phenomena overlap in the sorption process: polymer composition, nature of cross-linking, surface chemistry and morphology, porosity and tortuosity, inhomogeneity, etc. The DVS data obtained for pullulan–dopamine cryogels are depicted in Figure 6. The most relevant information that can be obtained is the material’s capacity to efficiently adsorb water molecules. As expected, the control sample, PO, exhibited the lowest adsorption efficiency, 31.41%, due to the smooth pores and the surface chemistry consisting of OH groups and hemiacetal cross-links. The best performance was recorded for the POD cryogel, 59.01%, which can be correlated with the rough pore surfaces observed in the SEM micrographs. Moreover, the addition of dopamine led to a surface with a large number of OH groups available to form hydrogen bonds with water molecules. Sample POD1 exhibited intermediate sorption, i.e., 40.71%, which corresponds to the SEM images.

The shape of the adsorption curve can provide additional information. The oxidized pullulan hydrogel, PO, exhibited a type III adsorption curve (BDDT classification). The isotherm exhibits a gradual increase of the adsorption volume with RH due to multilayered adsorption, capillary filling and capillary condensation. Such curves indicate that no saturation point will be reached. In contrast, the pullulan–dopamine hydrogels, POD and POD1, led to type II adsorption curves. These isotherms are characterized by a slow increase of the convex curve in the first half and a sharp increase in the second part of the isotherm. This type of curve is obtained for macroporous materials. The shift in trend may be an indication that the monolayer coverage is complete and the multilayer adsorption begins. The phenomena associated with the type II and III curves are similar. However, the major difference lies in the interactions between the polymeric surface and the water molecules, given by the presence of dopamine [41].

According to the IUPAC guidelines, all three hysteresis loops can be classified as type H3. Their shape is correlated to a specific pore structure. In this case, such curves are generated by materials with slit-shaped pores and panel-shaped particles. The desorption slope is associated with the force given by the tensile strength effect. However, a series of differences can be noticed: PO has a large sorption–desorption loop; in contrast, POD exhibited a very narrow cycle, while POD1 has an intermediate loop. In other words, PO induces a significant capillary condensation phenomenon. In contrast, in the POD hydrogel, there is little remnant water. This behavior can be explained by the presence of dopamine in the structure, leading to changes in both surface chemistry and internal morphology, i.e., protuberances, rough pore walls and newly formed filiform cross-links [41].

### 2.4. Swelling Behavior—Analysis of the Kinetics and Mechanism

**Figure 7:**
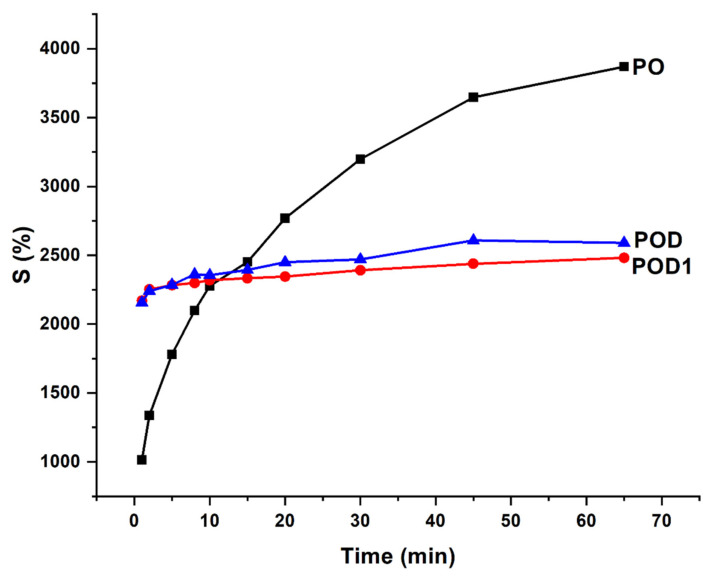
Swelling kinetic of the hydrogels in water.

When attempting to define hydrogels, one essential aspect to take into account is the materials’ ability to absorb and retain water and other fluids by diffusion, polymer relaxation or other mechanisms. Therefore, swelling experiments are of paramount importance to characterize such materials, regardless of their application. Hydrogel behavior in an aqueous medium was studied in Millipore water, and the results of these assays are plotted in Figure 7. Water with a high degree of purity was chosen to study the swelling phenomenon without the interference of ions, proteins, or other compounds.

The first observation that can be drawn from the kinetic studies is that the swelling degree of the PO hydrogel was significantly higher than those of hydrogels containing dopamine and was approximately 4000%. Furthermore, the swelling ratio of the PO sample exhibited a logarithmic increase and did not reach a swelling plateau. Therefore, the process tends to the dissolution of the polymer. In contrast, the POD and POD1 samples had fast initial swelling, followed by a stabilization phase marked by a moderate, slower process of water absorption. This is a clear indication of the fact that the addition of dopamine to the oxidized pullulan network entailed a significantly higher number of interactions between the polymeric chains. Dopamine caused not just a drop in the swelling degree, but also a notable reduction in the amount of time necessary for the swollen materials to reach stability. It can be assumed that changes in morphology, pore surface, and available moieties change with composition and preparation method.

Due to the substantial difference in the swelling behavior between the three materials, an in-depth analysis is required through a model fitting. The Korsmeyer–Peppas equation was chosen and fitted to the experimental kinetic data, and the results are listed in Table 1.

Firstly, the selected model is a very good fit for the PO hydrogel, with a correlation coefficient of over 0.99, compared to the hydrogels with dopamine, where the same parameter is around 0.95. Secondly, the swelling mechanism is correlated with the value of the exponent n, and four situations can be identified: *(i)* when *n* is less than *0.5*, the process is dominated by *Fickian diffusion*, and water movement is guided by a naturally occurring concentration gradient; *(ii)* when *0.5* < *n* < *1*, the *abnormal Fickian diffusion* is dominant, which means that water absorption is due to both water diffusion and relaxation of polymer chains; *(iii) n* = *1* the transport is primarily driven by the *macromolecular relaxation* of the polymer chains; and *(iv)* n > 1 the *abnormal Fickian diffusion* is no longer dominant, which means that the macromolecular relaxation and the erosion of polymer chains contribute together to the process of water absorption. In this situation, it can be concluded that Fickian diffusion of the water molecules is the predominant phenomenon in the swelling process because the diffusion coefficient n is less than 0.5. However, it is noteworthy that n values are 10 times smaller for POD than for PO.

### 2.5. Rheological Behavior

**Figure 8:**
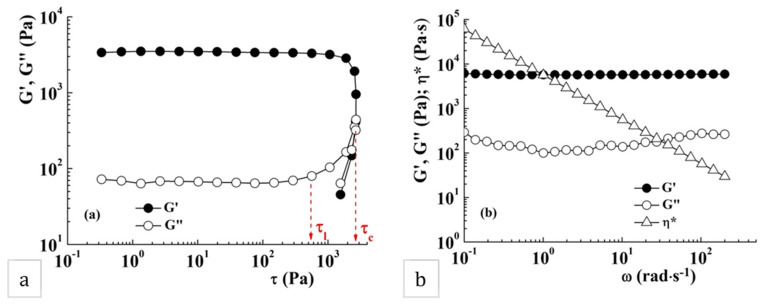
Viscoelastic properties of sample POD: (**a**) the linear viscoelastic range at 25 °C and 10 rad·s^−1^; (**b**) variation of G′, G″ and η* as a function of oscillatory frequency, ω, at 25 °C and 10 Pa.

The viscoelastic properties of the sample POD were evaluated and discussed. The amplitude sweep test reveals the gel-like behavior of the sample, with a storage modulus (G′) greater than the loss modulus (G″) (Figure 8a). The storage (G′) and loss (G″) moduli are independent of the shear stress up to a limiting shear stress (τ_l_), which corresponds to a limiting deformation. Further increase in shear stress determines the changing of the sample structure and, above a critical shear stress (τ_c_), the network structure is destroyed; G″ becomes higher than G′ and the sample acquires liquid-like properties. The network structure of the sample POD remains unchanged below 565 Pa, corresponding to a deformation of about 17%.

The gel-like behavior, where G′ values exceed those of G″, was also evidenced in the frequency sweep test (Figure 8b). The storage modulus, G′, remains constant on the whole domain of investigated oscillatory frequencies while the loss modulus, G″, shows a slight frequency dependence and a minimum value around 1 rad·s^−1^. This behavior is typical for the soft glassy materials, characterized by structural disorder and metastability, where the thermal motion is not sufficient to completely relax the structure [42]. The gel POD exhibits the viscoelastic moduli with about an order of magnitude higher than those reported for pullulan–polydopamine hybrid hydrogels [43].

**Figure 9:**
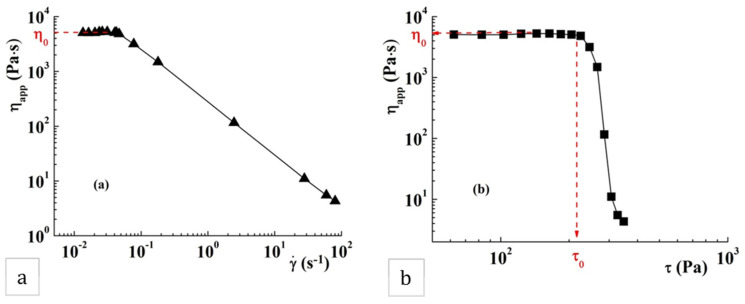
Variation of the apparent viscosity, η_app_, as a function of (**a**) shear rate ($\overset{\cdot }{\gamma })$ and (**b**) shear stress (τ) for sample POD at 25 °C.

The continuous shear tests reveal the pseudoplastic behavior of the sample POD, characterized by decreases in the apparent viscosity, η_app_, with the increase of the shear rate, $\overset{\cdot }{\gamma }$ (Figure 9a). The value of the zero shear viscosity, defined as the viscosity of material at zero shear rate (at rest), was determined to be 5144 Pa·s. The yield stress value, τ_0_, is an important rheological parameter of the material, and represents the minimum value of shear stress that can be applied to start material flow. In this sample, τ_0_, determined as the stress value at which the apparent viscosity abruptly decreases in the representation of η_app_ as a function of τ (Figure 9b), was about 220 Pa.

**Figure 10:**
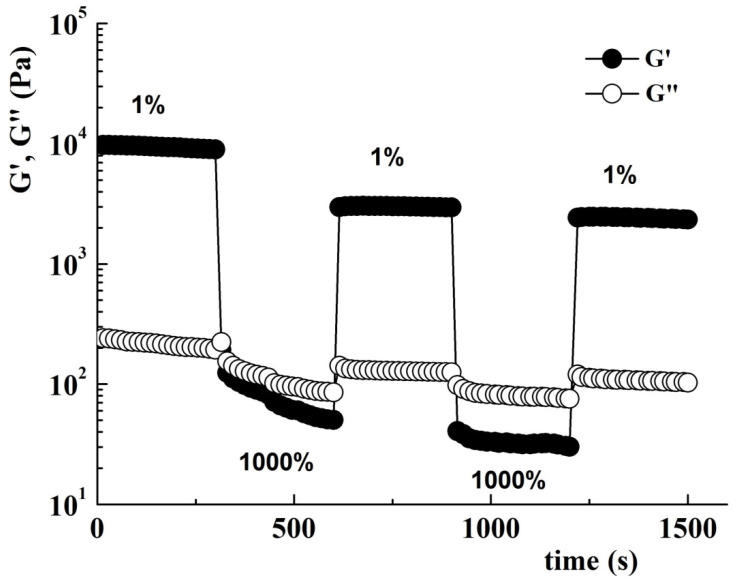
Variation of G′ and G″ when the strain is alternating from 1% to 1000% at 25 °C and 10 rad·s^−1^.

The structure recovery of sample POD was investigated at 10 rad⋅s^−1^ alternating low (1%) and high (1000%) strain in five consecutive cycles of 300 s duration (Figure 10).

In the cycles where the low deformation is applied, the sample preserves its gel properties with G′ > G″. At high strain, G′ and G″ are instantly diminishing and the sample acquires liquid-like properties. The loss tangent, tan δ (=G″/G′), is a measure of the ratio between the lost and stored energy during deformation cycle. When applying a high strain, the tan δ values increase as a result of the breaking of the hydrogel network. During the second cycle, when a strain of 1000% is applied, a decrease of G′ and G″ from 192 Pa and 224 Pa to 50 Pa and 85 Pa, respectively, was observed. In the second cycle with high deformation, the decrease was no longer observed, concluding that the breaking of the polymer network was finalized in the previous cycle with applied high strain. The gel recovers about 34% of its initial structure after the first high strain pulse and the final recovery is about 25%.

### 2.6. Mechanical Stability—Behavior under Compression Stress

**Figure 11:**
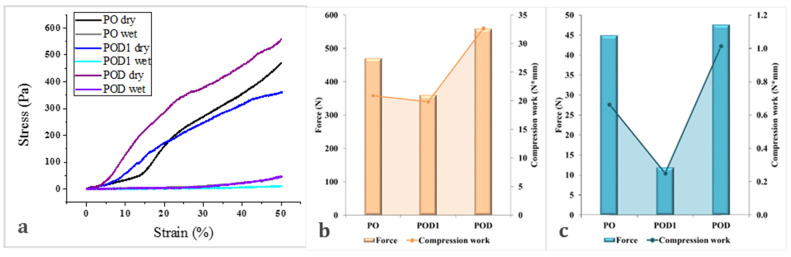
Mechanical properties of the cryogels: stress–strain curves (**a**) and illustration of the force and compression work for the materials in dry ((**b**), orange) and hydrated ((**c**), blue) states, respectively.

Since wound management, especially under acute, emergency circumstances, requires rapid intervention using reliable dressing materials, we chose to subject the prepared cryogels to a series of compressive tests. They were performed in both dry and hydrated states to evaluate their mechanical stability. The resulting stress–strain curves and the force and compressive work values are illustrated in Figure 11.

All materials exhibited excellent mechanical resistance when tested in the dry state, as indicated by the stress–strain curves and the values recorded for force and work. In contrast, the compressive force used on the hydrated samples is 10 times smaller. The mechanical work is even more strongly impacted by the fact that the cryogels absorbed water and became softer. Interestingly, no fracture signs were observed in the dry materials when subjected to a 50% compression load. Furthermore, all materials recovered in different degrees after the release of the load and even more so after hydration (data not shown).

The impact of the changes in composition can also be observed. The most promising results were obtained for POD. This sample exhibited a mechanical stability of up to 558 N. The other two samples, PO and POD1, had lower performances of 469 N and 360 N, respectively. The compressive work followed the same trend, with a maximum for POD, of 32.68 N mm. However, the values recorded for the compression work for PO and POD1 cryogels were very close (20.9 and 19.8 N mm). Similar results were obtained for the cryogels in the hydrated state, but some changes can be observed. POD remained the most mechanically stable sample, while POD1 was easily compressed and exhibited little resistance to the load. Furthermore, PO demonstrated an intermediate behavior and significantly higher compression work than POD1.

### 2.7. Hemocompatibility of the Pullulan–Dopamine Cryogels

The hemocompatibility of biomaterials is one of the essential criteria for their success in medical applications. Often after evaluating the hemocompatibility of some new medical devices, their applicability is drastically limited. Moreover, the favorable interaction between a material and the blood tissue is strongly contingent on composition, internal morphology, porosity, surface roughness and chemistry etc. [44,45,46,47].

**Figure 12:**
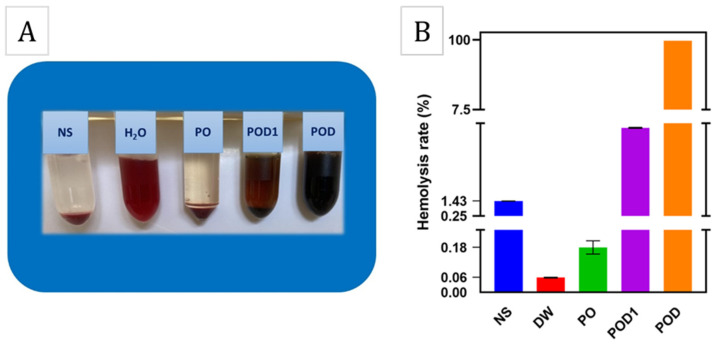
Hemocompatibility results: photographs of after the hemolysis test for the cryogel and control samples (**A**), and the hemolysis rate of the corresponding test and control samples (**B**).

The in vitro hemolysis results are shown in Figure 12. As can be observed, the three materials exhibit very different behavior when in contact with blood. The sample PO, based solely on oxidized pullulan, induced a similar effect as the negative control, i.e., the NS solution, where a colorless and clear supernatant was observed. In complete opposition, the POD and POD1 cryogels determined the presence of an intense red supernatant after centrifugation, much like the positive control which was the distilled water (sample DW). This result is a consequence of the fact that most of the red blood cells were ruptured and released the hemoglobin within. The hemolytic rates of the samples PO, POD1 and POD were 0.15%, 7.12% and 99.04%, respectively. These results may be explained by the adhesive nature of dopamine and the formation of numerous interactions between the material surface and the erythrocyte membrane, and may represent preliminary suggestions to the coagulant activity of these cryogels.

**Figure 13:**
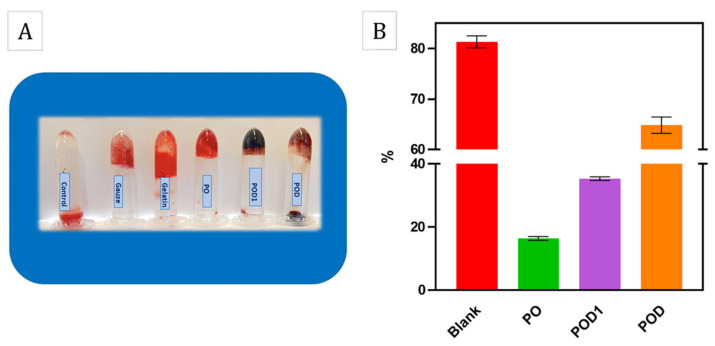
In vitro blood-coagulation results: (**A**) blood-coagulation photographs of control, medical gauze, gelatin and cryogel samples; (**B**) BCI values of the cryogel samples incubated in whole blood for 10 min.

For an additional hemostatic assessment, BCI is conventionally used as an effective quantitative test to demonstrate blood coagulation in vitro. The results shown in Figure 13B suggest that the cryogel samples have lower BCI values compared to the control which demonstrates their ability to participate in blood clotting. Figure 13A shows the blood fluidity variations after the treatments using gauze, gelatin, PO, POD1 and POD cryogels, respectively. These results may be due to the high blood absorption capacity of cryogel samples. In addition, with an increasing oxidation ratio, the BCI values decreased and the PO and POD1 samples gave the lowest values of 15.78%. It is already known that a lower BCI value is attributed to a better procoagulant effect of the hemostatic agent. According to the results obtained from the cryogel samples, we believe that PO had higher potential than the rest of the cryogels used in this study.

## 3. Conclusions

The starting hypothesis of this study was that stable cryogels can be obtained based on dialdehyde pullulan and physically entrapped dopamine. The data gathered from various characterizations point to the fact that hemiacetal groups alone are not sufficient, whereas the addition of dopamine and its chemical interaction with pullulan lead to a stable tree-dimensional network. Structural and morphological analysis confirmed the successful incorporation of the two components and the formation of porous matrices with internal morphology depending on the composition. The swelling exceeded 2000%, reaching an equilibrium in a matter of minutes. The rheological investigations revealed the obtaining of a pseudoplastic gel (G′ > G″) with a stable structure on the applied oscillation frequency range, characterized by high viscoelastic parameters. The gel exhibits zero shear viscosity and yield stress values of 5144 Pa·s and 220 Pa, respectively. The gel structure is destroyed under high shear (strain of 1000%), and when the shearing is stopped, only 25% of its structure is recovered. Compression tests indicated good mechanical resistance in dry state and a fine capacity of recovery. As demonstrated in the results, the cryogels had an obvious coagulation effect on whole sheep blood compared to the control. The results strongly point to the fact that the incorporation of dopamine into the dialdehyde pullulan matrix is paramount for the stability of the network. Moreover, the method used is another critical aspect when processing these hydrogels—dopamine must interact with the oxidized pullulan in solution, in order to allow the formation of a minimal necessary number of hemiacetal and imine bonds and stabilize the macromolecules. Therefore, this strategy leads to successfully obtaining hydrogels and may inspire new approaches to the design of hemostatic materials.

## 4. Materials and Methods

### 4.1. Materials

Prior to its usage, a pullulan sample with Mw = 100 kDa that had been bought from Carbosynth (San Diego, CA, USA) was dried overnight at 100 °C under vacuum. Dopamine hydrochloride and sodium periodate were purchased from Sigma-Aldrich (St. Louis, MO, USA) and used without prior purification. In addition, the other chemicals and solvents employed for pullulan oxidation were of a pure grade (Sigma-Aldrich, St. Louis, MO, USA) and did not undergo any further purification steps.

### 4.2. Cryogel Preparation

#### 4.2.1. Synthesis of Oxidized Pullulan

The first step in the preparation of the designed materials was to synthesize the oxidized pullulan derivative, enabling the formation of physical networks. To confirm our hypothesis, pullulan was oxidized by the periodate method [28]. Initially, 1 g of pullulan was solubilized in distilled water and then 0.2 g of sodium periodate was added. The periodate oxidation occurred at room temperature in the dark and the mixture was allowed to react for six hours. Afterwards, the solution was dialyzed for three days against distilled water, freeze-dried, and characterized or used for the preparation of cryogels.

#### 4.2.2. Preparation of Pullulan–Dopamine Cryogels

In order to confirm our hypothesis, we designed three types of cryogels: a pullulan control sample and two pullulan–dopamine materials, as follows. First, the control sample was based solely on oxidized pullulan (**PO**) and was obtained by freeze-drying a dialdehyde pullulan solution containing 1 g of polysaccharide. Second, two oxidized pullulan–dopamine cryogels were prepared using two different mechanisms of dopamine incorporation, i.e., either in the pullulan solution or in the freeze-dried pullulan scaffold. Therefore, 0.2 g of dopamine was added to an oxidized pullulan solution and the mixture was kept in contact for 2 h in order to allow the Schiff base reaction. Next, the solution was frozen and lyophilized, thus obtaining the **POD** cryogel. By comparison, a **POD1** cryogel sample was obtained by immersing a freeze-dried pullulan scaffold into a 0.2% dopamine solution in Millipore water. The mixture was kept in contact for 2 h, after which it was frozen and lyophilized. The amount of dopamine adsorbed by the POD1 cryogel was determined by means of UV-VIS spectroscopy (SPECORD 200 spectrophotometer, Analytik Jena, Jena, Germany).

### 4.3. Methods

#### 4.3.1. Nuclear Magnetic Resonance Spectroscopy

Nuclear Magnetic Resonance (NMR) spectra were obtained using a Bruker-Avance DR X 400 MHz Spectrometer (Bruker Corporation, Billerica, MA, USA). The instrument was outfitted with a 5 mm QNP direct detection probe with z-gradients. In order to record the ^1^H-NMR spectra, solutions of pullulan, dopamine and hydrogel in D_2_O were prepared. Tetramethylsilane (TMS) served as the internal standard at a concentration of 0 parts per million (ppm).

#### 4.3.2. Fourier-Transform Infrared Spectroscopy

Fourier-transform infrared (FTIR) spectra were obtained using an IRAffinity-1S spectrometer (Shimadzu Corp., Kyoto, Japan), coupled with dedicated IR software developed by LabSolutions (Shimadzu Corp., Kyoto, Japan). The scanning was performed in the range 4000 cm^−1^ to 400 cm^−1^, with a resolution of 4 cm^−1^. The spectra for all samples were obtained by scanning in transmission mode. The cryogels were frozen and plastered in order to prepare KBr pellets.

#### 4.3.3. Environmental Scanning Microscopy (ESEM)

Environmental scanning microscopy (ESEM, FEI Company, Thermo Fisher Scientific, Hillsboro, OR, USA) was used to investigate the internal morphology of the cryogels. Prior to scanning, the samples (40 mg cylindrical samples with a diameter of 15 mm and 5 mm height) were coated with a thin layer of gold. Environmental scanning equipment was used and each coated surface was analyzed with a 5 kV module with secondary electrons in high-vacuum mode. An LFD detector and an energy-dispersive spectroscopy system (EDAX, FEI Company, Thermo Fisher Scientific, Hillsboro, OR, USA) were both connected to allow additional elemental analysis of the investigated samples.

#### 4.3.4. Determination of the Apparent Density and Porosity of the Cryogel

The weight (*m*_0_), diameter (r•2) and height (*h*) of cylindrical cryogel samples were measured by means of analytical balance and digital caliper, respectively, aiming to determine the network density (*ρ_h_*). Classical mathematical equations were used in the calculations:

(1) $$\rho_{h}\left(\frac{mg}{cm^{3}}\right)=\frac{m_{0}}{V}$$

where *V* is the volume of cryogel that has been determined using the formula:

(2) $$V=\pi r^{2}h$$

The porosity of the cryogels was determined using the approach described prior in the literature [48,49] and the equation used was as follows:

(3) $$Porosity\;\left(\%\right)=\frac{m_{1}-m_{0}}{\rho V}\times 100$$

where the meanings of *m*_0_ and *V* in Formula (3) are the same as those in Formula (1). The weight of the cryogels after they have been soaked in ethanol is denoted by the symbol *m*_1_, and *ρ* is the density of ethanol (0.789 g/cm^3^). To summarize, an already-weighed cryogel was submerged in ethanol for 24 h, after which it was removed and reweighed. As a result of its high wettability and lack of any kind of cross-reaction with the cryogel, ethanol was selected for the task of filling the pores. In addition to that, it does not have the ability to disintegrate the cryogel or make it swell [50]. Each of the measurements of the samples was carried out thrice.

#### 4.3.5. Dynamic Water Vapor Sorption Studies

A fully automated moisture sorption analyzer IGAsorp (Hiden Analytical, Warrington, UK) was used for assessing the dynamic water vapor sorption capacity of the cryogels. Isotherms (at 25 °C) and kinetic curves, in a dynamic regime, were recorded, measuring the gravimetric sorption of water vapors of the freeze-dried samples, i.e., the change in weight with the variation of relative humidity (RH) in the range 0–60% in 10% increments for a full absorption/desorption cycle.

#### 4.3.6. Swelling Measurements

The swelling behavior of the hydrogels was determined by immersion in deionized Millipore water at room temperature. At specific time intervals, the samples were withdrawn from the swelling medium, the surplus liquid was readily absorbed with filter paper, and the samples were weighed. Based on the measurements, the swelling ratio (*S*) was determined using the following equation:

(4) $$S\left(\%\right)=\frac{m_{t}-m_{0}}{m_{0}}\times 100$$

where *m_t_* is the weight of the swollen sample and *m*_0_ is the initial weight of the dry sample. In order to get an accurate reading, each measurement was repeated three times and used as the mean average.

The kinetic data obtained (up to 60% of the maximum swelling capacity) were subjected to model fitting, using the power law expressed by Equation (5), in order to study the mechanism of the hydrogel swelling behavior.

(5) $$\frac{M_{t}}{M_{eq}}=Kt^{n}$$

where *M_t_* and *M_eq_* represent the quantity of solvent absorbed by the hydrogel at time *t*, respectively, at equilibrium, *K* is the swelling constant characteristic of the system, and *n* denotes the diffusion coefficient that relies on the nature of the solvent transport mechanism [51]. The values for the constants *n* and *K* were derived from the slope and intercepts of the plots of logarithmic (*M_t_/M_eq_*) vs. logarithmic (*t*) based on the experimental data.

#### 4.3.7. Mechanical Tests

Mechanical studies by means of compression were performed using a Shimadzu AGS-J deformation apparatus (Shimadzu, Columbia, MD, USA), at ambient temperature as follows. The freeze-dried sample was compressed to 50% at a deformation rate of 1 mm/min, with a load cell capable of measuring forces of up to 1 kN. After reaching the targeted level of deformation, the stress was released and the sample was allowed to recover and measured using a caliper. Subsequently, the cryogel was hydrated with 0.5 mL distilled water, subjected to a second compression at 50% deformation, and then allowed to recover and remeasured. Based on the stress–strain curves, various parameters were calculated and are presented and discussed in the following sections.

#### 4.3.8. Rheological Measurements

The rheological tests were realized on a MCR302 Anton-Paar rheometer (Graz, Austria) using plane–plane geometry with a diameter of 25 mm. The rheometer is equipped with a Peltier device for temperature control, and a solvent trap cover (Malvern Instruments Ltd., Worcestershire, UK) was used to limit solvent evaporation.

The linear viscoelasticity region (LVR) was determined by an amplitude sweep test at an angular frequency of ω = 10 rad⋅s^−1^, in the strain (γ) range of 0.01–1000% (the shear stress (τ) between 0.1 Pa and 3000 Pa). The storage (G′) and loss (G″) moduli, and the complex viscosity (η*) were determined by frequency sweep measurements performed between 0.1 rad·s^−1^ and 200 rad·s^−1^, at a shear stress of 10 Pa from LVR. The flow curves, zero shear viscosity (η_0_) and yield stress (τ_0_) values were determined by rotational measurements at shear rates ($\overset{\cdot }{\gamma }$) from 0.01 s^−1^ to 100 s^−1^.

The structure recovery after deformation was evaluated by an oscillatory step test at 10 rad·s^−1^ with five cycles of 300 s duration, in which low (from LVR) and high (from outside LVR) deformations are alternately carried out: 1%-1000%-1%-1000%-1%. All rheological measurements were performed at 25 °C on the sample POD (40 mg cylindrical samples with a diameter of 15 mm and 5 mm height) swollen in water for 15 min to reach equilibrium. The other samples, PO and POD1, were not suitable for rheological characterization, since they were not stable and disintegrated during the measurements.

#### 4.3.9. In Vitro Hemolysis Assays

For performing the in vitro hemolysis tests, sheep blood was collected and put in contact with a sodium citrate anticoagulant (1 mL), and then diluted to a concentration of 2% with a normal saline (NS) solution. The normal saline and deionized water were used as negative and positive controls, respectively. Prior to the experiments, weighted samples (25 g) of each of the three materials was immersed in 1 mL NS solution and kept at 37 °C for 12 h. After incubation, the diluted blood (100 μL) was added to the experimental and control groups and left in contact for one hour at 37 °C, then centrifuged at 3000 rpm/min for 10 min. The absorbance of the supernatant was measured at 545 nm with a microplate reader (Bio-Rad Laboratories Inc., Hercules, CA, USA).

The hemolysis rate was calculated by the following detection equation, previously described [32,52]:

(6) $$\mathrm{Hemolysis}\;\mathrm{rate}\;\left(\%\right)=\frac{{\mathrm{OD}}_{\mathrm{sample}}-{\mathrm{OD}}_{\mathrm{negative}}}{{\mathrm{OD}}_{\mathrm{positive}}-{\mathrm{OD}}_{\mathrm{negative}}}\times 100$$

GraphPad Prism version 8.0 (GraphPad Software Inc., San Diego, CA, USA) was used to perform the statistical analysis. The one-way analyses of variance were followed by Student’s tests, where *p* < 0.05 indicated statistically significant data and *p* < 0.01 indicated extraordinarily significant data.

#### 4.3.10. In Vitro Blood-Clotting Performance

Blood coagulation was evaluated by the blood-clotting test and reflected by the blood-clotting index (BCI), according to a previously reported method [53,54].

A total of 25 mg of PO, POD, and POD1 samples was separately put into conical flasks and then 100 μL of sheep citrate whole blood (activated by 0.2 M CaCl_2_) were added to the surface of each sample. As a control group, two types of commercial hemostatic materials (medical gauze and gelatin sponge) were treated with the same volume of activated sheep blood. Then, the tubes were incubated at 37 °C for 30 s, 1 min, 3 min, 5 min and 10 min. After the preset periods, 10 mL of deionized water were added carefully to all the test groups to release the unbound blood without disturbing the clot, and placed at 37 °C for another 10 min to rinse the uncoagulated blood.

Subsequently, the absorbance of the supernatant was measured at 540 nm with a microplate reader (Bio-Rad Laboratories Inc., Hercules, CA, USA). Three replicates were performed. For BCI the absorbance of 100 μL of whole blood mixed in 10 mL deionized water was used as the reference value (negative control).

The BCI was calculated using an equation previously described [32].

(7) $$\mathrm{Blood}\;\mathrm{clotting}\;\mathrm{index}\;\left(\%\right)=\frac{{\mathrm{OD}}_{\mathrm{sample}}}{{\mathrm{OD}}_{\mathrm{reference}}}\times 100$$

## Figures and Tables

**Table 1:** Plots illustrating the goodness of the fit of the Korsmeyer–Peppas equation on the experimental data and the resulting parameters: the correlation coefficient (**R^2^**), the exponent (**n**) and the Korsmeyer–Peppas constant (**k**).

Sample Code	PO	POD	POD1
**Simulation plot**	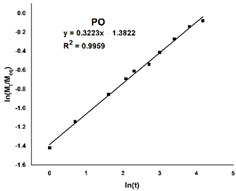	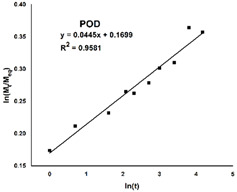	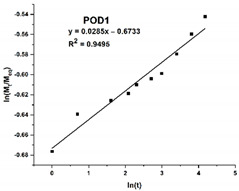
**R^2^**	0.9959	0.9581	0.9495
**n**	0.3223	0.0285	0.0445
**k**	0.2511	0.5101	1.1852

## Data Availability

Not applicable.
